# Risk and protective factors for burnout among physicians from standardized residency training programs in Shanghai: a cross-sectional study

**DOI:** 10.1186/s12913-020-05816-z

**Published:** 2020-10-21

**Authors:** Lei Huang, Jennifer Harsh Caspari, Xiaoting Sun, Jessica Thai, Yaxi Li, Fa-zhan Chen, Xu-dong Zhao

**Affiliations:** 1 Medical Education Division & Department of Psychiatry, Tongji Hospital, Tongji University School of Medicine, Shanghai, China; 2 Tongji University School of Medicine, Shanghai, China; 3 Department of Internal Medicine, University of Nebraska Medical Center, Omaha, NE USA; 4 College of Medicine, University of Nebraska Medical Center, Omaha, NE USA; 5 Shanghai Pudong New Area Mental Health Center, Tongji University School of Medicine, Shanghai, China; 6 Shanghai East Hospital Affiliated to Tongji University, Shanghai, China

**Keywords:** Burnout, Residents, Standardized residency training program, Occupational stress, Social support, Empathy, Work situations, Survey and questionnaires

## Abstract

**Background:**

High burnout has been reported in physician populations. Although the standardized residency training (SRT) in China includes components that might put residents at a higher risk for burnout, the burnout of Chinese medical residents is unknown. This study aimed to evaluate the prevalence of burnout and the associated risk and protective factors for medical residents in the SRT program in Shanghai, China.

**Methods:**

This study was a prospective cross-sectional design. A random sampling strategy was used to recruit 330 resident physicians from four SRT sites in Shanghai, and 318 completed questionnaires were returned. Respondents completed a self-made questionnaire including demographic and work characteristics, four burnout and wellness-specific surveys. Bivariate analyses and hierarchical multiple regression models were used to analyze factors associated with three sub-scales of burn out separately.

**Results:**

The overall burnout rate was 71.4%. Low level rate of personal accomplishment (PA) was extremely high at 69.5%. Night shift experience, high occupational stress, and low social support were significant predictors, which explained 49.1% variance of emotional exhaustion (EE) (F = 26.528, *P* < 0.01). Factors that significantly predicted depersonalization (DP) included male gender, senior residents, night shift experience, high occupational stress, and low psychological empathy, which explained 51.5% variance totally (F = 29.004, *P* < 0.01). Senior residents, high income, low occupational stress, and high empathy were also significant predictors of decreased personal achievement (PA), which explained 18.4% variance totally (F = 12.897, *P* < 0.01).

**Conclusions:**

There was a high burnout rate among SRT residents in Shanghai. Occupational stress and several work-related factors were significant and strong risk factors for burnout, while empathy and social support were mild protective factors. Decreased work-related demands and increased access to resources could assist residents in reducing their work stress and improving their well-being.

## Background

Burnout is a syndrome characterized by perceptions of excessive demands, lack of enthusiasm, and feelings of frustration or cynicism due to a reduced sense of accomplishment [1]. The most widely used three-component model of burnout is by Maslach, which includes emotional exhaustion (EE), depersonalization (DP), and feelings of decreased personal accomplishment (PA) [2]. Burnout can lead to many challenges, including a physician’s mental health issues such as suicidal tendencies and physical symptoms such as fatigue and headaches, as well as health system troubles such as poor doctor-patient relationships and clinical malpractice [3–6]. Furthermore, the negative consequences of burnout for physicians in training include reductions in job satisfaction and work effort, high job turnover, and patient safety [6–8]. As a result, burnout among physicians has drawn global attention.

Burnout is a common experience for medical residents due to long working hours, little control over daily events, and high levels of responsibility [9–11]. A literature review showed that a burnout rate among residents was 27–75%(depending on specialty) [12]. Another study on U.S. residents and fellows revealed the burnout rate of 60.3% [13]. Echoing with the findings in western literature, doctors who are younger than 40 years old had a higher prevalence of burnout than senior physicians in China [14]. A systematic search of databases conducted for papers published from 2006 to 2016 showed that the overall burnout rate of doctors in China was between 66.5–87.8%, reaching an epidemic level [14]. Studies focusing on residents indicated that 54.99–78.3% of the respondents experienced burnout syndrome [15–17]. These alarming figures may suggest an underestimated problem below the tip of the iceberg.

A lot of factors contribute to the risk of burnout among medical workers in China [12]. Regarding the sociodemographic factor, doctors of younger age were shown a higher level of burnout. Studies indicated that the prevalence of burnout was significantly higher among doctors under the age of 35 [18], the score of exhaustion was significantly higher in the group of 30–40 years age than that in any other groups [19]. Unmarried physicians and work with less experience were easy to expose to higher burnout risk compared with doctors on the contrary [18, 20]. What’s more, family income and work-family conflict were associated with burnout among Chinese doctors [21]. Regarding the occupational stress caused by work-related factors, including experience career uncertainty, low salaries, and a very heavy workload due to China’s large population are highlighted [22]. Some physicians in China see 70–80 patients in one day [23]. Furthermore, Chinese physicians work within a medical system where the patient-doctor relationship has significantly deteriorated to the point that thousands of doctors have been physically injured by dissatisfied patients [24]. Previous burnout studies have demonstrated that Chinese physicians faced many challenges causing some to lose their enthusiasm for their career and to regret choosing to study medicine because of relatively poor compensation and challenging working environments [25].

In addition to these challenges, a new standardized residency training (SRT) program for medical trainees in China was initiated nationally. This new program was designed to enhance the quality of medical services offered in China and to create more standardization in physician training. Hospitals in Shanghai were the first to pilot this program since 2010 [26]. One of the largest changes in Shanghai was the switch of trainee designations from “unit persons” to “society persons.” Residents must find employment after training often in different hospitals or cities. This transition brings inherent challenges such as employment uncertainty and less attachment to the training sites for SRT residents. In addition, SRT also adds three years of high-stress training and relatively low work compensation [27]. With the additional stressors, burnout rates may be increased for these trainees compared to trainees who are not taking part in the SRT program.

Conversely, there are several protective factors related to burnout, which have assisted medical residents reduce their risk of burnout. Empathy has been defined as “a predominantly cognitive attribute that involves an understanding of experiences, concerns, and perspectives of the patient” [28]. Studies have evidenced that empathic engagement in patient care leads to improved patient satisfaction and clinical outcomes [29, 30], which may help to prevent or reduce burnout. A previous study evidenced that high empathy was significantly associated with low burnout [31–33] and reducing professional burnout could help keep medical workers’ empathy levels high [34]. As a result, empathy may be a tool to help address the burnout of SRT residents in China. Social support is based on an individual’s assessment of the support available in a given situation [35]. The harmful impact can be significantly decreased if a source of support available in a stressful situation [36]. Social relationships might help residents mitigate the deleterious effects of burnout. It is suggested that hospitals can potentially help promote interventions that stabilize and nurture social relationships for the medical trainees [37, 38]. A study on medical workers in southwest China showed social supports lightly affect the level of burn out syndrome [39].

To our knowledge, the majority of published studies on residents’ burnout were conducted before the national SRT program was initiated [17, 40]. Studies focused on the residents in SRT programs are limited. However, residents in SRT programs in China are facing different challenges than those in previous training programs and their job demands and resources have changed. Because of this, it is critical to collect new data from studies to understand the burnout of residents’ in the SRT programs accordingly. Most of the studies on the burnout of Chinese residents in SRT programs often utilized convenience sampling with respondents from only one training site [41] or one specialty [42]. Demographic and work-related predictive factors were usually discussed [43] but the use of multiple standardized instruments as predictive factors were limited. Furthermore, previous studies focused on the risk factors for burnout [41, 44, 45], while largely failing protective factors, including social support and empathy were not discussed in the previous studies on physicians in the SRT program. To fill this research gap, our study tried to enroll two protective factors to comprehensively predict burnout outcomes of residents from the SRT program. Additionally, Shanghai was the first city that the SRT program was established and is the only city where all residents from the SRT program are “society persons.” Residents in other cities, even those in SRT, are more frequently able to retain employment in the same hospitals for training than those in Shanghai. Therefore, Shanghai SRT residents are more similar to other international trainees such as US residents, excluding some interfering factors caused by job uncertainty. This current study was designed (1) to evaluate the prevalence of burnout for medical residents in the SRT program in four training sites in Shanghai with a random sampling design, and (2) to better understand risk and protective factors for burnout among Chinese medical residents in the SRT program using three standardized scales in a multivariate statistical model.

## Methods

### Study setting

The study was conducted in four hospitals in Shanghai, including three comprehensive hospitals and one specialty hospital for women and children. The hospitals are SRT training sites certified by the Shanghai Municipal Health Commission.

### Research design and conceptual framework

This study was a prospective cross-sectional design employing survey techniques. There are several models to explain the phenomena of burnout. One commonly cited model is the Job Demands Resource (JDR) model, which proposes that working conditions can be categorized into job demands and job resources. Lower levels of job resources and higher levels of job demands predict greater burnout [36]. In this model, occupational stress and work-related conditions are considered as the most important job demands, while sources and functions of social support, autonomy, and opportunities for professional development are significant factors of job resources [17, 37, 39]. Our study employed JDR model and hypothesized occupational stress and work-related conditions to be risk factors, and empathy and social support as protective factors in the final model to better predict burnout outcomes.

### Sampling and sample size

We employed a random sampling technique by inputting all the badge numbers of the residents undergoing training at the time of the survey in June 2017 into a random generator computer program. The sample size for the study was determined using the formula of single population proportion formula. Based on the previous related study reported on the burnout prevalence rate of Chinese residents (76.7%) [17], a 95% confidence level, and margin of error (0.05) and calculation, the minimum sample size of 275 was required. Trainees were stratified according to their departments and year of training, and half of the residents within each stratification were randomly selected by the computer program and contacted by program assistants. Out of 330 residents selected, a total of 318 residents (141 males and 177 females) completed the survey with a response rate of 96.4%.

### Data collection tool and data collection

The data was collected using a self-administrated survey consisting of four standard scales, demographic, and work situation items. Before the massive distribution of the survey, we conducted a pilot study by inviting 20 residents from one institution to complete this survey, in order to modify the wording to ensure that all questions were easily understood. A member of the study core team was present at the time of filing the survey to clarify questions as needed.

#### Outcome -- burnout

Burnout was measured using the Chinese version of the Maslach Burnout Inventory-Human Services Survey (MBI-HSS) [46], which was translated from English [47] and revised to make the items culturally and linguistically applicable to Chinese respondents. The Chinese version of the MBI-HSS has been widely used among Chinese medical professionals [42, 44]. The questionnaire includes 22 items with three dimensions: nine items for emotional exhaustion (EE), five items for depersonalization (DP), and eight items for personal accomplishment (PA). The cut-off points of the MBI-HSS in the Chinese version were: EE: low≤16, moderate 17–26, high ≥27; DP: low ≤6, moderate 7–12, high ≥13; PA: low≤31, moderate 32–38, high ≥39. Individuals with high levels of EE, high levels of DP, or low levels of PA were identified as having high levels of burnout [48]. In this study, Cronbach’s α for EE, DP, and PA was 0.860, 0.827, and 0.893, respectively. Scores of each MBI-HSS subscale were used as continuous outcome variables in identifying the predictive factors separately.

### Predictive factors--occupational stress, empathy, social support, work conditions and demographics

Occupational stress was measured by the Chinese Physician’s Job Stressor Scale (CPJS). The scale was developed with consideration for Chinese culture and has been used among Chinese health professionals [49, 50]. It consists of 25 items measuring five dimensions: career development (score range 5–25), workload and time allocation (4–20), work environment and resources (3–15), patient care (8–40), and interpersonal relationships (5–25). Each item has a 5-point Likert scale and possible total scores range from 25 to 125. Higher scores indicate greater stress. The scale has been validated with a Cronbach’s α coefficient of 0.909. The five dimensions were 0.846, 0.808, 0.717, 0.776, and 0.793, respectively.

Residents’ empathy was measured using the Chinese version of Jefferson Scale of Physician Empathy (JSPE). It has been specifically modified for use in medical students, with promising results supporting the use of this modified version amongst a variety of health care professions [51, 52]. The JSPE contains 20 questions, 10 of which are negatively worded and reversely scored. It is delivered via a 7-point Likert scale, where subjects must respond to a statement with options ranging from strongly disagree to strongly agree. It consists of three dimensions including “perspective-taking,” “compassionate care,” and “standing in the patients’ shoes.” Possible total scores range from 20 to 140. Higher scores indicate a greater behavioral tendency toward empathetic engagements during patient care episodes [28]. The Cronbach’s alpha coefficients for the overall scale was 0.750. The split-half reliability and the test-retest reliability were 0.771 and 0.659, respectively [53].

Social support was measured via the widely used Social Support Rating Scale (SSRS) [54–56]. This scale was congruent with the Chinese culture and is comprised of three dimensions: objective support, subjective support, and support utilization. Objective support reflects respondents’ social networks and the tangible, instrumental, and emotional support that they have received in the past. Subjective support refers to perceptions of being respected, supported, and understood [57]. Support utilization reflects the extent to which individuals seek and make use of social support. Total SRSS scores range from 12 to 65, with a higher score indicating a higher level of social support. The scale has been validated with a Cronbach’s α coefficient of 0.89–0.94 and a test-retest reliability of 0.92 [58].

Work situations included work experience before SRT, year in SRT, night shift experience, weekly working hours, daily sleeping hours, annual income, satisfaction with annual income, and attitude towards the necessity of the SRT. Because there was an overlap in July when our survey was carried out, the year in SRT was divided into the categories of “just entering”, “at the end of 1^st^ year”, “at the end of 2^nd^ year”, and “at the end of 3^rd^ year”. Night shift experience referred to whether residents worked the night shift most recently in the last month. Satisfaction with annual income was scored as “unsatisfied”, “neutral”, and “satisfied”. Attitude towards the perceived necessity of the SRT was delineated into “unnecessary”, “neutral”, and “necessary”.

Demographics including age, gender, marital status, and education were also collected. Marital status was classified as “unmarried/divorced/widowed” or “married/cohabitation”. Education referred to the highest medical degree level upon entering the SRT, including “bachelor’s degree”, “master’s degree”, and “doctoral degree” .

### Data analysis

Respondents’ demographics, work situations were summarized using descriptive statistics (numbers and frequencies). Each burnout dimension was categorized into mild, moderate, and high, in order to define if the respondent suffered from high burnout. Bivariate analyses were performed between each of the three burnout dimensions and predictive variables. Specifically, two independent sample t-test or one-way ANOVA was used accordingly to examine the mean differences of the outcomes varied by the categorical factors. Hierarchical multiple regression was employed to select variables that were responsible for the largest proportion of the explained variance. Variables that showed significance in bivariate analyses were kept in the Hierarchical multiple regression. The significance level was set at 5%. SPSS version 19.0 (IBM Corporation) was used for all analyses.

### Ethical consideration

The name was not required to file in the survey in order to protect privacy. This project received approval from the Ethics Review Committee of Tongji Hospital of Tongji University (Registration Number KYSB-2016-100).

## Results

### Characteristics of respondents and burnout prevalence

A total of 318 respondents returned completed questionnaires and were enrolled in final analyses. Respondents were from different specialties of four training sites. A total of 73 (23.0%) residents were from Shanghai Tenth People’s Hospital, 146 (45.9%) from Shanghai Tongji Hospital, 77 (24.2%) from Shanghai East Hospital, and 22 (6.9%) from Shanghai Maternity & Infant Hospital. Seventy-nine (24.8%) respondents were from internal medicine, 59 (18.6%) were from surgery, 41 (12.9%) were from obstetrics & gynecology, 27 (8.5%) were from family medicine, 25 (7.9%) were from medical imaging & nuclear medicine, 23 (7.2%) were from neurology, 19 (6.0%) were from emergency medicine, and the remaining 45 (14.2%) respondents were from psychiatry, pediatrics, medical laboratory, ophthalmology, anesthesiology, otorhinolaryngology, rehabilitation, and dermatology, with each specialty making up less than 4% of the total respondents. Detailed about other demographic and work characteristics of respondents were in Table 1.

**Table 1:** Demographic and work characteristics of residents (*n* = 318)

Variables	Number	Percent
**Gender**		
Male	141	44.3
Female	177	55.7
**Marital Status**		
Unmarried/Divorced/Widowed	254	79.9
Married/Cohabitation	64	20.1
**Work experience before SRT**		
None	214	67.3
Yes	104	32.7
**Education**		
Bachelor’s degree	200	62.9
Master’s degree	99	31.1
Doctoral degree	19	6.0
**Year in SRT**		
Just entering	77	24.2
At the end of 1st year	106	33.3
At the end of 2nd year	85	26.7
At the end of 3rd year	50	15.7
**Night shift**		
No	24	7.5
Yes	294	92.5
**Satisfaction with annual income**		
Unsatisfied	168	52.8
Neutral	119	37.4
Satisfied	31	9.7
**Recognition on necessity of SRT**		
Unnecessary	59	18.6
Neutral	105	33.0
Necessary	154	48.4

Respondents with high levels of EE and DP and low levels of PA were 45 (14.2%), 41 (12.9%) and 221 (69.5%), respectively. Because the low level of PA was predominant among our respondents, we used the definition of burnout by Elmore [48]. The total burnout rate was calculated by counting the number of respondents having “high EE,” “high DP,” or “low PA.” A total of 227 (71.4%) respondents met these criteria for “burnout” (Table 2).

**Table 2:** Prevalence of different categories of burnout measured using MBI-HSS

Levels	EE		DP		PA	
	N	%	N	%	N	%
Mild	137	43.1	154	48.4	221	69.5
Moderate	136	42.8	123	38.7	63	19.8
High	45	14.2	41	12.9	34	10.7

*MBI-HSS* Maslach Burnout Inventory-Human Services Survey, *EE* Emotional Exhaustion, *DP* Depersonalization, *PA* Personal Accomplishment

### Bivariate analysis between each burnout measure and predictive factor

The results of the descriptive analysis for respondents’ demographics and work situations and the comparisons of the mean difference of three burnout dimensions across categorical groups are displayed in Table 3. There was a significant difference between males and females for EE (t = 2.562, *P* = 0.012) and DP (t = 3.552, *P* < 0.001). “Married/Cohabitation” residents reported higher EE scores compared to those who were “unmarried/divorced/widowed” (t = − 2.039, *P* = 0.045). Residents who had work experience before SRT reported higher EE (t = − 3.349, *P* = 0.001) and DP (t = − 4.403, *P* < 0.001) scores than those who did not work before entering SRT. Increasing resident year in SRT was positively associated with EE(F = 7.507, *P* < 0.001) and DP (F = 13.727, *P* < 0.001) scores. Residents who were just entering or almost finished with SRT had higher PA scores compared to those in their second and third years of training (F = 4.366, *P* < 0.001). Residents who worked night shift in the last month reported higher EE (t = − 3.225, *P* = 0.001) and DP (t = − 3.545, *P* < 0.001) scores compared with those who did not. Respondents who were more satisfied with the annual income had lower EE (F = 19.535, *P* < 0.001) and DP scores (F = 15.62, *P* < 0.001) and higher PA scores (F = 3.909, *P* = 0.021). Respondents who agreed with the necessity for the SRT program had lower EE (F = 15.111, *P* < 0.001) and DP (F = 20.121, *P* < 0.001) scores.

**Table 3:** Bivariate analysis of different categories of burnout with demographic and work characteristics of residents

Variables	EE			DP			PA		
	Mean ± SD	F/t	P	Mean ± SD	F/t	P	Mean ± SD	F/t	P
**Gender**									
Male	19.89 ± 10.25	2.526	0.012	8.38 ± 5.40	3.552	< 0.001	27.71 ± 8.11	0.392	0.695
Female	17.47 ± 6.74			6.50 ± 4.00			27.36 ± 7.66		
**Marital Status**									
Single/Divorced/Widow	17.96 ± 7.84	−2.039	0.045	7.01 ± 4.28	−1.980	0.051	27.59 ± 7.56	0.338	0.735
Married/Cohabitation	20.86 ± 10.69			8.63 ± 6.17			27.22 ± 8.98		
**Work experience before SRT**									
None	17.44 ± 7.45	−3.349	0.001	6.54 ± 3.91	−4.403	< 0.001	27.75 ± 7.47	0.755	0.451
Yes	20.81 ± 10.13			8.97 ± 5.83			27.04 ± 8.59		
**Education**									
Bachelor degree	17.97 ± 8.22	1.715	0.182	6.97 ± 4.49	1.669	0.19	27.99 ± 7.59	0.987	0.374
Master degree	19.16 ± 8.92			8.03 ± 5.15			26.74 ± 8.34		
Doctor degree	21.32 ± 9.64			7.53 ± 5.14			26.58 ± 7.95		
**Year in SRT**									
Just entering	14.91 ± 6.20	7.507	< 0.001	4.74 ± 2.99	13.727	< 0.001	29.99 ± 8.17	4.366	0.005
At the end of 1st year	18.65 ± 8.38			7.30 ± 4.42			26.23 ± 7.89		
At the end of 2nd year	20.44 ± 9.47			8.65 ± 5.06			26.41 ± 8.04		
At the end of 3rd year	20.68 ± 8.81			9.16 ± 4.47			28.32 ± 5.92		
**Night shift**									
No	13.21 ± 5.57	−3.225	0.001	4.08 ± 3.27	−3.545	< 0.001	29.54 ± 8.03	1.316	0.189
Yes	18.98 ± 8.61			7.60 ± 4.76			27.35 ± 7.83		
**Satisfaction with annual income**									
Unsatisfied	21.22 ± 9.29	19.535	< 0.001	8.67 ± 5.32	15.620	< 0.001	26.36 ± 7.59	3.909	0.021
Neutral	15.55 ± 6.66			5.71 ± 3.48			28.81 ± 7.99		
Satisfied	15.48 ± 5.70			6.29 ± 3.52			28.81 ± 8.01		
**Recognition on necessity of SRT**									
Unnecessary	23.81 ± 11.65	15.111	< 0.001	10.61 ± 6.47	20.121	< 0.001	26.24 ± 8.38	1.973	0.141
Neutral	17.63 ± 6.69			7.03 ± 4.11			26.97 ± 7.75		
Necessary	17.14 ± 7.51			6.29 ± 3.77			28.38 ± 7.66		

*SRT* Standardized Residency Training, *SD* Standard Deviation, *EE* Emotional Exhaustion, *DP* Depersonalization, *PA* Personal Accomplishment. F/t stands for the bivariate analysis parameter. Specifically, t value was reported if two independent sample t-test was used. F value was reported if one-way ANOVA was used

### Hierarchical multiple regression analyses

The results of the hierarchical linear regression analyses of the factors influencing MBI-HSS subscale scores are shown in Tables 4, 5 and 6. For EE, only night shift, occupational stress, and social support showed significant results in the final model with an adjusted R^2^ value of 0.491. Night shift experience, high occupational stress, and low social support predicted a high level of EE (F = 26.528, *P* < 0.01). For DP, gender, year in SRT, night shift experience, occupational stress, and empathy were included in the final model with an adjusted R^2^ value of 0.515. Male, senior residents, night shift experience, high occupational stress, and low psychological empathy predicted a high level of DP (F = 29.004, *P* < 0.01). For PA, year in SRT, annual income, occupational stress, and empathy were included in the final model with an adjusted R^2^ value of 0.184. Senior residents, high income, low occupational stress and high empathy predicted a high level of PA (F = 12.897, *P* < 0.01).

**Table 4:** Hierarchical multiple regression of emotional exhaustion (EE)

Variables	Model1	Model2	Model3	Model4	Moedel5
**Demographics**					
Age^(^yr)	0.066	−0.084	− 0.053	− 0.053	−0.050
Gender	−0.135*	− 0.079	− 0.064	− 0.059	− 0.053
Marital Status	0.108	0.096	0.050	0.054	0.087
**Work situations**					
Work experience before SRT		0.093	0.025	0.026	0.022
Years of SRT		0.146**	0.077	0.065	0.046
Night shift		0.142**	0.140**	0.140**	0.129**
Daily sleep hours		−0.140**	− 0.014	− 0.009	0.020
Satisfaction with annual income		−0.242**	−0.065	− 0.068	−0.058
Recognition on necessity of SRT		−0.143**	−0.026	− 0.018	−0.010
**Occupational pressure**			0.597**	0.583**	0.547**
**Empathy**				−0.055	−0.016
**Social support**					−0.185**
Δ*R*^*2*^	0.041	0.231	0.484	0.486	0.511
*Adjusted R*^*2*^	0.032	0.208	0.467	0.468	0.491
*F*	4.471**	10.254**	28.763**	26.313**	26.528**

* P < 0.05, **P < 0.01*;* Demographics and work situations were controlled in the mode. In step1, demographics were added. In step2, work situations were added. In step3, occupational pressure was added. In step4, empathy was added. In step5, social support was added

**Table 5:** Hierarchical multiple regression of depersonalization (DP)

Variables	Model1	Model2	Model3	Model4	Moedel5
**Demographics**					
Age (yr)	0.103	−0.089	−0.070	−0.067	−0.061
Gender	−0.184*	−0.126**	− 0.114**	−0.100*	− 0.096*
**Work situations**					
Work experience before SRT		0.132*	0.068	0.071	0.072
Years of SRT		0.198**	0.146**	0.113*	0.103*
Night shift		0.142**	0.142**	0.141**	0.135**
Daily sleep hours		−0.140**	−0.031	−0.015	0.000
Annual income (×10^4^ RMB)		0.080	0.033	0.041	0.044
Satisfaction with annual income		−0.205**	−0.029	−0.040	− 0.036
Recognition on necessity of SRT		−0.198**	−0.094*	− 0.074	−0.070
**Occupational pressure**			0.545**	0.508**	0.493**
**Empathy**				−0.151**	−0.132**
**Social support**					−0.085
Δ*R*^*2*^	0.049	0.299	0.509	0.528	0.533
*Adjusted R*^*2*^	0.043	0.278	0.494	0.511	0.515
*F*	8.096**	14.58**	31.886**	31.065**	29.004**

* P < 0.05, **P < 0.01*;* Demographics and work situations were controlled in the mode

In step1, demographics were added. In step2, work situations were added. In step3, occupational pressure was added. In step4, empathy was added. In step5, social support was added

**Table 6:** Hierarchical multiple regression of personal achievement (PA)

Variables	Model1	Model2	Model3	Model4
**Work situations**				
Years of SRT	−0.013	0.028	0.102	0.113*
Annual income (×10^4^ RMB)	0.175**	0.074	0.091	0.088
Satisfaction with annual income	−0.180**	− 0.146**	−0.161**	− 0.165**
**Occupational pressure**		−0.265**	−0.167**	− 0.143*
**Empathy**			0.313**	0.286**
**Social support**				0.102
Δ*R*^*2*^	0.053	0.110	0.191	0.199
*Adjusted R*^*2*^	0.044	0.098	0.178	0.184
*F*	5.835**	9.63**	14.739**	12.897**

* P < 0.05, **P < 0.01*;* Work situations were controlled in the mode

In step1, work situations were added. In step2, occupational pressure was added. In step3, empathy was added. In step4, social support was added

The effects of variables on the variance in MBI-HSS subscale scores are displayed. Demographic variables explained 4.1% of the variance in EE and 4.9% of the variance in DP. Work situations accounted for an additional 19.0% of the variance in EE, 25.0% of the variance in DP, and 5.3% of variance in PA. Occupational stress explained 25.3% of the variance in EE, 21.1% of the variance in DP, and 5.7% of the variance in PA. Empathy was responsible for 0.2, 1.8, and 8.1% of the variance in EE, DP, and PA, respectively. Social support accounted for 2.5, 0.5, and 0.8% of the variance in EE, DP, and PA, respectively.

## Discussion

This study evaluated the prevalence and factors associated with medical residents’ burnout among the newly implemented SRT program in China. Our respondents were randomly sampled from four SRT hospitals in Shanghai, which is the pilot city to carry out this national residency training program. Our findings on the predictive factors, especially firstly explored empathy and social support as protective factors for residents’ burnout may direct the improvement of SRT programs in Shanghai, which could have a great impact throughout China since the SRT programs implemented in Shanghai have now been launched in numerous other Chinese cities since 2015.

In our sample, the overall burnout rate was 71.4%. This is consistent with a literature review from China, in which researchers found that physicians’ burnout rates range from 66.5–87.8% [14]. However, this rate of burnout was higher than the burnout rate for US general surgery residents (69.0%) using the same scale and definition [48]. The variance in prevalence rates of burnout from study to study may be related to the use of different definitions, assessment instruments, and cutoff criteria [12, 59]. As a result, it is difficult to directly compare burnout rates among studies. However, another study on Chinese SRT residents showed a burnout rate of 62.2% [41], higher than a study conducted with residents pre-SRT (54.99%) with the same scale [15]. Although it is challenging to determine if the burnout rates for Chinese residents increased following SRT implementation, the high burnout rates in our current study suggest a concerning issue that may be unaddressed in many residents and training programs.

Furthermore, compared to EE and DP, nearly 70% of residents showed extremely low levels of PA, which is very similar to the findings of previous studies on Chinese residents SRT in the program [44, 60]. The result showed that low level of PA was predominant syndrome of burnout among our respondents. However, study from U.S. general surgery residents revealed that only 16% of residents scored in the lowest tertile of PA [48]. Several reasons may explain the difference. Firstly, in comparison with young doctors in the U.S., the residents in China are extremely unsatisfied with their salaries [49]. A study revealed that the median salary of Chinese doctors working in the secondary and tertiary hospitals of big cities is only about 5000 RMB (US$780) per month [61]. Salaries of residents of the SRT program are even lower than the average level. Secondly, Chinese residents spend a lot of paperwork instead of learning useful skills during the training [22], which may affect the improvement of their competency of patient care and decreased their self-confidence. Finally, misunderstandings and distrust between providers and patients [61] may increase a lot of work pressure [49] and reduced the accomplishment for the young residents.

Job Demands Resource Model (JDR) is the conceptual framework to explain the phenomena of burnout in our study. The result showed that factors related to work situations explained 19.0% of the variance in EE, 25.0% of the variance in DP, and 5.3% of the variance in PA. Year in SRT, night shift experience, and annual income were three significant factors related to burnout. Working night shift was a work-related risk factor associated with burnout in our study. Residents required to work nights reported higher scores of EE and DP. This is consistent with previous study results, which found that residents on night shift often face long duty hours, which would affect their job performance, increase medical errors, and reduce job satisfaction [62]. Long duty periods for residents in SRT programs have also been positively associated with burnout [41]. Interestingly, annual income was a negative predictor of PA. A possible explanation for this result is that residents with higher income may also have higher job demands, and their current income level may not satisfy their expectations [49, 50]. Moreover, residents in later years of training experienced higher levels of DP yet higher scores of PA, illustrating that “the year in SRT” was a complicated factor for burnout. According to the JDR theory, relationships between demand and work engagement are highly dependent on the nature of the demand. Demands that employees tend to categorize as challenges are positively associated with engagement [63]. Thus, although senior residents have more challenging tasks that that may lead to overload and loss of enthusiasm, it also can increase their sense of accomplishment. Consequently, it may be beneficial for both trainees and patients if SRT training sites limit duty hours and develop strategies to support residents in their final years of training in improving enthusiasm and overcoming lassitude.

Occupational stress was a significant risk factor for the three dimensions of residents’ burnout, which has been evidenced among Chinese doctors [64]. Factors commonly causing occupational stress were also prevalent in SRT residents in Shanghai such as high workload, low pay, and intense patient-physician relationships [15]. These factors could all contribute to the high burnout rate in SRT residents compared to other study populations. Furthermore, job uncertainty was a prominent feature of this study sample from Shanghai, who will most likely have to seek employment outside their training hospitals. However, most medical residents in China were “unit persons” prior to SRT implementation, which ensured the lifelong employment in the same hospital. This has been a huge change in job security for SRT residents [27]. In fact, previous studies demonstrated that medical students had a negative attitude towards SRT because of the career uncertainty following the residency [65]. This job uncertainty adds to occupational stress, which has been found to be highly associated with burnout among physicians [66]. Social support and empathy acted as mild but significant protective factors for experiencing burnout for our sample of residents, which is consistent with previous studies [32, 38]. Social support is often associated with more confidence, greater life satisfaction, and enthusiasm for work. In fact, greater perceived social support is associated with a lower risk for burnout. Greater peer-based social support has been associated with decreased personal and work-related burnout scores since this type of support decreases loneliness [37]. Studies on other occupations showed that burnout symptoms were related to perceived social support. Employees reported that a lack of social support from supervisors, colleagues, and the social community at work significantly increased burnout [58, 59]. Specifically, a study conducted on Chinese physicians confirmed that strong social support at work could reduce the risk for burnout [39], which is consistent with the findings of this current study. However, our data indicated that the relative contribution of social support and empathy on reducing the risk for burnout may not be significant. One possible explanation for this is that social support, especially from colleagues and supervisors, may be associated with work engagement, which will increase job demands in addition to providing support. Another study with residents from Singapore found similar results and showed that empathy and burnout may be manifestations of the same emotional constructs, which complicates their relationship with each other [33]. Furthermore, social support from the work environment and physicians’ empathy for patients was relatively lower for Chinese physicians compared to physicians from western countries [67], which may also contribute to the limited effects of social support in this study.

The findings of this study illustrate the potential benefit of incorporating empathy-promoting activities into the residency training curriculum. This aspect of patient care and provider well-being is often neglected in current SRT programs in China. Empathy training may help trainees improve their interpersonal and relationship-building skills for patient care and may assist with increased job satisfaction, which may lead to reductions in stress and burnout [32, 68, 69]. Additionally, training sites could assist trainees by providing strong social support for trainees via available consultation services, assigning mentors, or building up support groups through which trainees can share their experiences and support one another.

The additional challenges inherent in the SRT and the extremely high burnout rates experienced by Chinese SRT residents are concerning, and there is an urgent need to create resolutions to make training programs sustainable and effective for trainees. If burnout rates continue at their current rate, residents, patients, and quality of care may all suffer. Those who are responsible for creating and directing training programs can consider which pieces of residency training may be modified to reduce burnout. Examples include assisting residents by enhancing job security following residency and increasing work compensation during residency. Furthermore, if empathy and social support are protective factors for burnout, program leaders should develop programs within residency training that promote them. Examples include resident support groups such as Balint groups, in which residents process their experiences on the physician-patient relationship and comprehensive wellness programs that are built into the residency curriculum and attend to the biopsychosocial-spiritual wellbeing of residents.

### Limitations

The current study has several limitations. Firstly, it is a cross-sectional study, and the data collected from the respondents only reflect perspectives measured at that point. No causal relationships can be asserted between the examined factors and the outcomes. Secondly, the data was collected through self-administrated surveys. Hence, results are subject to response biases. For example, respondents with more positive attitudes and better resilience to adverse events in life may report fewer burnout symptoms and higher ratings on job satisfaction. This was accounted for with measurement tools embedded with questions designed to minimize these biases. Thirdly, for the dimension of PA, the contribution of predictive factors in this study was limited, and more related factors should be added to the model. Finally, the participant sample was from only four training sites in Shanghai, which limited the applicability of the results to the broader Chinese population. Future research may repeat this study with a larger and more representative sample from training sites in other cities.

## Conclusions

This current study found that Chinese medical residents in the SRT program in Shanghai experienced a high level of burnout, especially in the domain of low PA. Occupational stress was found to be the strongest indicator of burnout among Chinese residents. Work situations such as night shift, year in SRT, and annual income also had significant influences on burnout. Empathy and social support were mild but significant protective factors for burnout. Our results highlighted the importance of reducing job demands and improving resources to prevent burnout. Efforts should be made to reduce work-related stress and to enhance mitigating factors for burnout such as social support and empathy, thereby decreasing residents’ burnout among the SRT program in China. However, the limited sample size of residents of the SRT program may limit the applicability of this study. Furthermore, due to this study’s cross-sectional study design and use of different burnout measurement instruments compared to other studies, we cannot firmly conclude that burnout rates increased after SRT program initiation. A cohort study with a larger sample size may further clarify whether burnout rates truly increased following SRT implementation.

## Data Availability

The data analyzed under the current study are available from the corresponding author on reasonable request.

## Abbreviations

SRT: Standardized Residency Training;
MBI-HSS: Maslach Burnout Inventory-Human Services Survey
EE: Emotional Exhaustion
DP: Depersonalization
PA: Personal Accomplishment
CPJS: Chinese Physician’s Job Stressor Scale
JSPE: Jefferson Scale of Physician Empathy
SSRS: Social Support Rating Scale
